# Length–mass allometries of the larvae of aquatic dipterans: differences between taxa, morphological traits, and methods

**DOI:** 10.1093/jisesa/ieae012

**Published:** 2024-02-17

**Authors:** Julien Mocq, Vladimíra Dekanová, David S Boukal

**Affiliations:** Faculty of Science, Department of Ecosystem Biology & Soil and Water Research Infrastructure, University of South Bohemia, Branišovská 1760, CZ-37005 České Budějovice, Czech Republic; Czech Academy of Sciences, Biology Centre, Institute of Entomology, Branišovská 1760, CZ-37005 České Budějovice, Czech Republic; Faculty of Science, Department of Ecosystem Biology & Soil and Water Research Infrastructure, University of South Bohemia, Branišovská 1760, CZ-37005 České Budějovice, Czech Republic; cE3c – Centre for Ecology, Evolution and Environmental Changes & CHANGE – Global Change and Sustainability Institute, Faculty of Sciences of the University of Lisbon, Edifício C2, Campo Grande, 1749-016 Lisbon, Portugal; Faculty of Science, Department of Ecosystem Biology & Soil and Water Research Infrastructure, University of South Bohemia, Branišovská 1760, CZ-37005 České Budějovice, Czech Republic; Czech Academy of Sciences, Biology Centre, Institute of Entomology, Branišovská 1760, CZ-37005 České Budějovice, Czech Republic

**Keywords:** length–weight regression, dry mass, wet mass, Culicidae, mosquito

## Abstract

Body mass underpins many ecological processes at the level of individuals, populations, and communities. Often estimated in arthropods from linear morphological traits such as body length or head width, these relationships can vary even between closely related taxa. Length–mass relationships of mosquito (Diptera: Culicidae) larvae are poorly known despite the importance of this family to disease and aquatic ecology. To fill this gap, we measured ontogenetic changes in linear traits (body length, head width, and thorax width) and dry and wet masses and estimated length- and width–mass relationships in larvae of 3 culicid species inhabiting different niches: the tropical *Aedes albopictus* (Skuse, 1894), the temperate *Culex pipiens* (Linnaeus, 1758), and the snowmelt *Ochlerotatus punctor* (Kirby, 1837). We compared our results with published length–mass allometries of other aquatic dipteran larvae. We showed that thorax width and body length, but not head width, reliably predicted body mass for our 3 species. The length–mass allometry slopes in aquatic dipterans varied considerably between and within families but were independent of phylogeny, specimen handling, preservation techniques, and data fitting methods. Slope estimates became less precise with decreasing sample size and size range. To obtain reliable estimates of the allometric slopes, we have thus recommended using data on all larval stages for intraspecific allometries and a wide range of species for interspecific allometries. We also cautioned against the indiscriminate use of length–mass allometries obtained for other taxa or collected at lower taxonomic resolutions, e.g., when using length–mass relationships to estimate biomass production at a given site.

## Introduction

Body mass is a master trait that underpins life histories, trophic interactions, seasonal population patterns, distribution of resources, energy fluxes in food webs, and community structure in aquatic ecosystems (Burgherr and Meyer 1997, Woodward and Hildrew 2002, Giustini et al. 2008, Mährlein et al. 2016). Reliable body mass data are thus indispensable for the ongoing efforts to move from taxon-based to fully trait-based approaches in aquatic ecology (Usseglio-Polatera et al. 2000), however, directly measured body mass values are not always available in the literature.

There are 3 main methods widely used to estimate the individual biomass of invertebrates: direct weighing, estimation of biovolume, and relationships that link one or more linear morphological traits to the body mass (hereafter “length–mass relationships”; Burgherr and Meyer 1997). Since direct weighing is inaccurate for small individuals (Johnston and Mathias 1993) and sensitive to the manipulation of the sample (Leuven et al. 1985, Sabo et al. 2002) and biovolume estimation often underestimates body mass of larger individuals (Burgherr and Meyer 1997), many studies rely on length–mass relationships, commonly referred to as “length–weight relationships”. These relationships are usually expressed as allometries (see Eq. 1 in Materials and Methods; Johnston and Cunjak 1999, Sabo et al. 2002) and allow for more comprehensive comparisons of invertebrate populations within and between habitats and ecosystems (Benke 1993). They are thus widely used in ecological studies of freshwater macroinvertebrates (Burgherr and Meyer 1997, Benke et al. 1999, Johnston and Cunjak 1999, González et al. 2002).

Nonetheless, the number of published length–mass relationships varies considerably between taxa, with (almost) no data for some groups, including mosquitoes (Diptera: Culicidae). Members of this family are an important part of aquatic and terrestrial food webs (Mogi 2007, Quiroz-Martínez and Rodríguez-Castro 2007, Louca et al. 2009, Kraus and Vonesh 2012), in nutrient cycling (Walker et al. 1991, Mouquet et al. 2008), and are vectors of pathogenic viruses, protozoa, and nematodes (Hubálek 2008, Jourdain et al. 2019) including mosquito-borne diseases that are of great importance for human health (Mayer et al. 2017). It is, therefore, surprising that the mass dependence of the processes listed above and the Culicidae length–mass relationships are very patchily documented and poorly understood (Oliphant and Hyslop 2020).

To help fill this gap, we quantified the length–mass relationships in the larvae of 3 common mosquito species from the subfamily Culicinae occupying 3 contrasting niches: the tropical *Aedes albopictus* (Skuse, 1895), the temperate *Culex pipiens* (Linnaeus, 1758), and the snowmelt *Ochlerotatus punctor* (Kirby, 1837). *Aedes albopictus* is intensively studied owing to its invasive status and range expansion northward in Europe (Cunze et al. 2016, Ryan et al. 2019) and North America (Leisnham et al. 2008, Medley 2010, Rochlin et al. 2013) driven by climate change. It is an important vector of multiple diseases, including Zika, dengue, chikungunya, and West Nile arboviruses (Hubálek 2008). *Culex pipiens* is a widespread Holarctic species and a vector of the West Nile virus (Becker et al. 2010); the ongoing climate change will likely increase the geographical range of *C. pipiens* and the virus (Hongoh et al. 2012). The role of the widespread Holarctic species *O. punctor* as a disease vector is unknown (Martinet et al. 2019). We included this species because its larvae develop in cold-water ponds produced by melting snow and ice in forest swamps in early spring (Becker et al. 2010), and its different habitat and morphology could be reflected in different length–mass relationships. To put these data in a wider perspective, we also compiled previously published length–mass allometries of aquatic dipterans, which were compared to our own allometries, and analyzed if the slope of the length–mass allometry depends on phylogeny, ecological traits (such as current and thermal preferences), and 2 key confounding factors: the linear trait(s) used to estimate body mass and the methods used to obtain and analyze the data.

Most previous studies relied on one a priori chosen linear trait and rarely compared multiple traits in length–mass relationships (but see Wenzel et al. 1990, Becker et al. 2009, Shahbaz-Gahroee et al. 2021). Here, we compared 3 commonly used traits: head width, body length, and body width (= thorax width in mosquito larvae). Because of its strong sclerotization impeding within-instar growth, the head width is often a poorer estimator of body mass than body width or length (Dyar 1890). Moreover, dry and wet mass have been used in length–mass relationships, each with its respective advantages and disadvantages. Wet mass measurements are sensitive to the effectiveness of blotting and desiccation, and the error increases when individuals are pooled during the measurement process (Dermott and Paterson 1974). On the other hand, aquatic ecologists prefer to use wet mass (Edwards et al. 2009) to keep the individuals alive (Meyer 1989, Towers et al. 1994), e.g., when individuals are measured repeatedly over time. In addition, specimen preservation (especially in ethanol) can dissolve enzymes and lipids, affecting individual mass and soft morphological features (Leuven et al. 1985, Mährlein et al. 2016) and making measurements from preserved individuals potentially biased (Johnston and Cunjak 1999, Benke and Huryn 2006, Mährlein et al. 2016). We thus compared the dry and wet mass-based slopes of length–mass relationships in the 3 mosquito culicid taxa and in the whole dataset on aquatic dipterans. We assumed that the dry content increases with size (as in, e.g., Chironomidae: Dermott and Paterson 1974), leading to steeper slopes of the length–mass relationship based on dry weight. Finally, both length and mass are often measured with error, and their relationship can be treated as symmetrical, with no a priori explanatory and response variable. This favors the use of standardized major axis (SMA, also known as reduced major axis; Warton et al. 2006) regression instead of ordinary least squares (OLS) linear regression in length–mass allometric curve fitting (Smith 2009). We thus compared both methods when analyzing our data on the mosquito larvae.

Based on the arguments summarized above, we expected to observe (i) different allometric slopes for the 3 mosquito species and other aquatic dipterans that could be linked to taxon-specific differences in morphological traits, (ii) a better estimation of body mass (i.e., higher proportion of explained variance) by the thorax width or body length in the 3 mosquito species, and (iii) a significant effect of the measure of body mass (i.e., dry or wet mass), preservation method, and the method used to analyze the data (i.e., OLS vs SMA regression) on the allometric slope or intercept estimates across aquatic dipterans. More specifically, we expected shallower allometric slope in more streamlined, elongated species living in running waters (such as Ceratopogonidae and Simuliidae) and in allometries based on individuals preserved in ethanol if later instars have larger lipid stores that dissolve in ethanol (Mills et al. 1982). In addition, we expected that (iv) the length–mass allometry estimates improve (i.e., the 95% confidence intervals of the parameter estimates decline and/or the proportion of explained variance increases) with sample size and larger range of body sizes. We also expected an increase in 95% confidence intervals of estimates based on wet mass because we considered corresponding measurements less reliable due to the difficulty in standardizing the blotting among individuals.

## Materials and Methods

### Animal Husbandry and Collection

*Aedes albopictus* and *C. pipiens* were raised in the laboratory at the Biology Centre, Institute of Entomology in České Budějovice, Czech Republic. Eggs of both species were provided by the European project Infravec2 (project #3245) in 2019. The eggs were immediately placed in aged tap water at 20 °C upon delivery. After hatching, larvae of each species were kept together in 10 L containers in temperature-controlled rooms (18L:6D photoperiod) at ca. 25 °C (mean ± SD = 24.7 ± 0.3 °C). They were fed 3 times a week with grounded Pangamin Klasik (Brewer’s yeast *Saccharomyces cerevisiae* enriched with malt extract, Rapeto, Czech Republic) at a concentration of ca. 100 mg L^−1^, enabling the highest expected survival and growth (Romeo-Aznar et al. 2018). We removed excess food and refilled the containers with clean water once a week. After pupation, individuals were collected and placed with other conspecific pupae in rearing cages with netting (80 × 60 × 50 cm). Emerged adults were fed with a permanently available 15% glucose solution, and the females were also fed warm bovine blood on a cotton pad 3 times a week. For oviposition, *C. pipiens* females had access to 200 mL containers with aged tap water, and *A. albopictus* females had access to a coffee filter immersed in a 100 mL container with clean water without refill to let the water level decrease over time. Eggs of *C. pipiens* and *A. albopictus* were collected daily and once a week, respectively.

*Ochlerotatus punctor* larvae were collected in a swamp next to the village of Dobročkov, Czech Republic (48°55ʹ6.608″N, 14°9ʹ36.124″E) in early March 2019. Some of them were measured immediately, with emphasis on first-instar (hereafter L1) larvae. The remaining individuals were maintained in 10 L plastic containers in thermal cabinets (Lovibond BSK ET 650; Tintometer GmbH, Dortmund, Germany) at 6 °C (mean ± SD = 6.1 ± 0.2 °C) with 9L:15D photoperiod and fed with grounded Pangamin Klasik as above. Individual larvae were randomly sampled for measurements (see below), and some of them were raised to adults for identification.

### Trait Measurements

Individuals were randomly chosen for measurements from individuals in the maintenance containers at different times to cover all larval instars. We measured the total body length (*TL*; from the tip of the head to the base of the siphon), maximum thorax width (*TW*), and head width across eyes (*HW*) of each individual with QuickPhoto 3.1 software (Promicra, Czech Republic) from pictures taken with an Infinity1 camera (Lumenera, Canada) mounted on an Olympus SZX10 stereomicroscope. Each individual was then gently blotted on a slightly wetted paper towel to remove excess water and immediately weighted in a predried and preweighted aluminum cup on the MSA6.6S-0CE-DM microbalance (Sartorius, Germany) to determine the wet mass *W* to the nearest 0.001 mg. Afterward, the individuals were oven-dried at 50 °C for 24 h following (Benke and Huryn 2006) and weighed again using the same micro-balance to determine the dry mass (*DW*). While larvae of *O. punctor* were large enough to be weighed individually, early instar larvae of *C. pipiens* and *A. albopictus* had to be pooled to obtain reliable data (dry mass of L1 *C. pipiens* larvae: 10 pools of up to 6 individuals; wet and dry mass of L1 *A. albopictus* larvae: respectively 8 and 5 pools of up to 10 individuals, wet and dry mass of L2 *A. albopictus* larvae: respectively 30 and 17 pools of up to 3 individuals). Individuals from a pooled sample were assigned the same mass, which was calculated by dividing the total mass by the number of individuals. All individuals were kept alive until the weighting of the fresh mass and were not preserved using ethanol or other chemicals.

### Data Analyses

In length–mass relationships, individual dry or wet body mass (hereafter *M*) is usually linked to a morphological trait (hereafter *T*), usually the body length (hereafter *L*) through the allometric function:


$$\begin{array}{l} M=aT^{b} \end{array}$$ (1)

where *a* is the intercept and *b* is the allometric slope (Burgherr and Meyer 1997). This can be log-transformed to a linear relationship, usually using a natural or base-10 logarithm:


$$\begin{array}{l} ln\left(M\right)=ln\left(a\right)+b\;ln\left(T\right); \end{array}$$ (2a)

$$\begin{array}{l} log_{10}\left(M\right)=log_{10}\left(a\right)+b\;log_{10}\left(T\right). \end{array}$$ (2b)

Length–mass relationships assume that the individual dry or wet body mass varies predictably with one or more morphological traits, usually the total body length. The length–mass relationship is most often described by an allometry. The residual error in this equation is usually multiplicative, making nonlinear fitting inadequate. The allometry can be log-transformed to a linear relationship, typically using the natural (e.g., Pélabon et al. 2018) or decimal (e.g., Warton and Weber 2002) logarithm. Log-transformation of the data makes the error additive and usually equalizes the variance across the length range, which yields sufficiently precise and reliable estimates of the allometric slope from the linear regression of data (see Eq. 2 in Materials and Methods; Mährlein et al. 2016).

To compare SMA and OLS regression, we first regressed the log_10_-transformed wet and dry mass against each of the 3 log_10_-transformed linear traits using Eq. (2b) with SMA regression to estimate the trait- and response-specific intercept *a* and slope *b*. SMAs were fitted using Huber’s *M*-estimator to alleviate the bivariate contamination (i.e., down-weigh the outliers) and improve inference (Taskinen and Warton 2013). For each combination of mass and linear trait, we tested if the SMA slopes of the trait-mass regressions of all 3 Culicidae species were equal using likelihood ratio tests and compared the species-specific slopes using pairwise comparisons with *P-*values adjusted for multiple comparisons using the Dunn–Šidák correction. SMA elevations were compared between species with Wald statistics (Warton et al. 2012). The same datasets were then regressed with the OLS method. The back-transformation to raw body mass scale requires a correction against the underestimation of smaller individuals, such as the Duan’s smearing factor *SF*, used for Eqs. (2a) and (2b):


$$\begin{array}{l} SF=\frac{1}{n}.\underset{i=1}{\overset{n}{\sum }}\;e^{\varepsilon_{i}} \end{array}$$ (3)

where *n* is the number of data points, and *ε*_*i*_ are the regression residuals. This factor is applied as a multiplicator after the back-transformation of individual log-transformed masses to untransformed ones (Mährlein et al. 2016). We calculated *SF* for each trait-mass combination and each species for both regression methods. The higher the *SF* value, the higher the mass underestimation and, thus, the mass correction.

For each species and type of mass, we compared the fit of the SMA models to identify the linear trait among the 3 predictors total body length, head width, thorax width that fits best the data using *R*^2^ as the goodness-of-fit measure. We did not test for “significance” of the differences between predictor traits because the models were not nested. Finally, we compared the intercept *a* and the allometric slope *b* and their 95% confidence interval of the SMA and OLS regressions among the 3 species.

### Overview of Published Length–Mass Relationships in Aquatic Diptera

We compiled published estimates of allometric slopes of the length–mass relationships of *aquatic dipterans* using the search term (“Diptera* AND (Mass* OR Weight) AND (Length OR Size) AND (Aquatic OR Freshwater”)) in the Web of Science and Google Scholar as of January 2023, and selected only data reporting the slope between dry or wet mass and any linear morphological trait in aquatic dipterans. This yielded data on 219 allometries, including our own data on *A. albopictus*, *C. pipens*, and *O. punctor*. The following information was extracted: the deepest taxonomic level defined in the paper (i.e., from order to species), type of allometry (interspecific or intraspecific), larval stages included in the dataset (specific larval stages or entire ontogeny), fitting method (linear or nonlinear regression), type of body mass (dry or wet), linear trait (body length, head width, interocular width, and thorax width), minimum and maximum trait value, size range (as the difference between the log_10_-transformed minimum and maximum), slope value and its standard error, coefficient of determination of the relationship (*R*^2^), use of fresh or preserved individuals (i.e., if the individuals were processed fresh or preserved), preservation method (freezing, ethanol, and formaldehyde), and sample size.

We also searched relevant trait databases (Barbour et al. 1999, Tachet et al. 2000, Merritt et al. 2008, Schmidt-Kloiber and Hering 2015, Serra et al. 2016, Phillips and Smith 2018) and journal articles for functional traits of the taxa covered in our dataset and potentially influencing the length–mass relationships. Due to many missing values, we collated only data on current preference (as an ordered factor), feeding type (categorical), and locomotion type (categorical; Table 1). This procedure yielded data for 59 species or genera in 16 families (allometries: *N* = 145). When the functional trait included affinities with several categories, the category with the strongest affinity was selected, and in the case of multiple high affinities, we selected the average category or created a mixed category to represent the taxon trait.

**Table 1.** Ecological traits and their categories used as predictors in the length–mass allometries

Trait	Categories
Current preference	Limnobiontic/ Limnophilic/ Rheo-limnophilic/ Rheophilic/Rheobiontic
Feeding type	Active-filter feeders/ Passive-filter feeders/ Gatherers/ Grazers/ Predators/ Shredders
Locomotion type	Burrowing/ Sessile/ Sprawling/ Swimming

Finally, we assessed factors influencing the slope estimate and the width of its 95% confidence interval (calculated as 1.96 times the standard error of the estimate) of published length–mass relationships using generalized linear mixed-effect models (GLMMs). We used Gaussian-family GLMMs that were appropriate for the model residuals. In the “baseline” GLMM model, we included fitting method, standardized sample size, intra/interspecific allometry, standardized size range, and preservation method as main fixed effects without interactions, and the original source paper as a random effect to account for possible lack of independence among the results of analyses of multiple datasets in the same paper, e.g., due to undocumented differences in the details of the experimental and statistical methods. The slope estimate was added as a predictor in the models with a 95% confidence interval.

In the first analysis, family was used as an additional main fixed effect predictor in the baseline model. We used a model selection approach and compared their respective corrected Akaike information criterion (AICc) values to identify the most parsimonious model. AICc was used to avoid potential overfitting. Family was strongly correlated with several ecological traits and thus removed from the subsequent analysis in which we tested the possible effect of these ecological traits on the allometric slope and its confidence interval. We first tested the collinearity of the ecological traits by checking the variance inflation factor for quantitative factors and correlations for qualitative ones. Feeding type was the only trait exhibiting high collinearity and thus was removed. We again used model selection to compare the baseline model with the models that extended the baseline model by including locomotion, current preference, and both ecological traits. The most parsimonious model in each of these analyses was identified using AICc, and models with ΔAICc ≤ 2 were considered as plausible (Arnold 2010).

Finally, we tested if the allometric slope estimates depend on the phylogeny. Dipteran phylogeny used in the analysis was based on Wiegmann et al. (2011) and Lifemap (de Vienne 2016), and for specific taxonomic groups, Cranston et al. (2012), Krosch et al. (2017), and Krosch et al. (2022) for Chironomidae; Becker et al. (2010) for Culicidae; Ribeiro (2008) for Tipulomorpha; Evans and Adler (2000), Gil-Azevedo and Coscarón (2020), and LaRue et al. (2009) for Simuliidae, using the highest taxonomic resolution available in the paper (Supplementary Fig. S1). The phylogenetic tree was included without branch lengths in the analysis. The average slope of each taxon in the dataset (with slope estimates at the level of the entire order excluded) was used as the response variable to quantify a phylogenetic signal in the body length–mass and the head width–mass relationships, for which we had enough data. This signal was determined by the values of Moran’s *I* calculated by Abouheif’s test (Abouheif 1999, Pavoine et al. 2008) using Monte Carlo simulation (*N* = 999 randomizations) for each of the 2 relationships.

All analyses were performed in R version 4.1.2 (R Development Core Team 2019) using the packages *reshape2* (Wickham 2007), *dplyr* (Wickham et al. 2023), *smart* (Warton et al. 2012), *lme4* (Bates et al. 2015), *multcomp* (Hothorn et al. 2008), and *emmeans* (Lenth 2024) for data manipulation and analyses; *picante* (Kembel et al. 2010), *ape* (Paradis and Schliep 2019), *adephylo* (Jombart et al. 2010), and *ggtree* (Yu et al. 2017) for phylogeny analysis and visualization; and *ggplot2* (Wickham 2016) and *patchwork* (Pedersen 2024) for graphics.

## Results

### Body Mass and Length–Mass Allometries of the 3 Mosquito Species

**Fig. 1:**
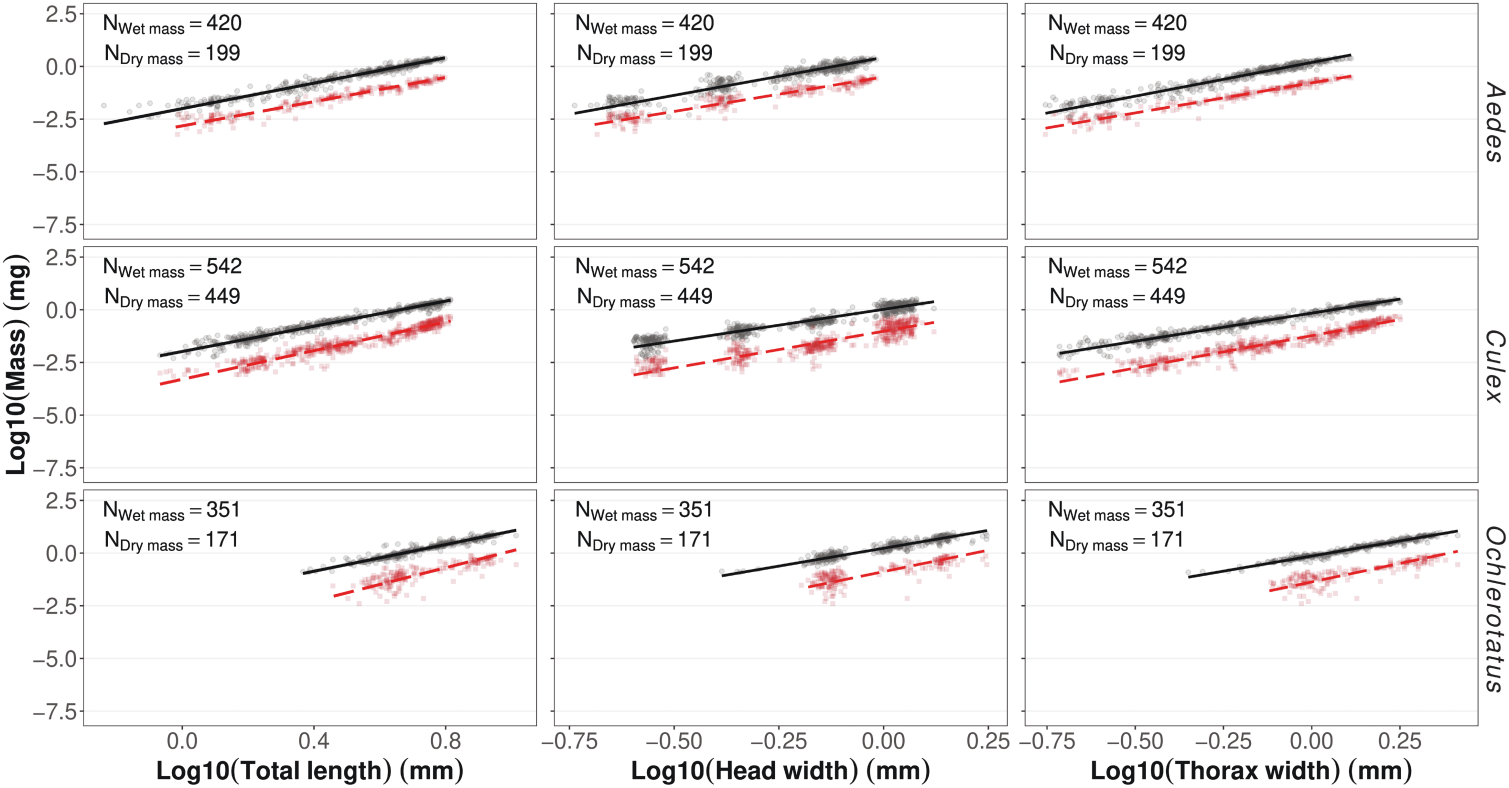
Individual body mass and linear traits (points) and estimated length–mass relationships (based on SMA regressions, Eq. 2b; lines) for the 6 possible intraspecific length–mass allometries for the 3 mosquito species. Red squares and dashed line = dry mass; black circles and solid line = wet mass; sample sizes for each type of mass are given in each panel. Individual body mass and linear traits and estimated length–mass relationships based on SMA regressions for the 6 possible intraspecific length–mass allometries for the 3 mosquito species Aedes albopictus, Culex pipiens and Ochlerotatus punctor

*Ochlerotatus punctor* was systematically larger and heavier than *C. pipiens* and *A. albopictus* of the same larval stage, while the latter 2 were often similar in size and mass (Fig. 1, Supplementary Table S1).

Elevation values ln(*a*) from the SMA regressions differed significantly in all 3 species for the *TL-DW* and *TW-W* allometries. For the other 4 trait-mass combinations, the elevation values did not significantly differ between *O. punctor* and *A. albopictus* (*HW-W* allometry), between *A. albopictus* and *C. pipiens* (*TL-W* allometry), or between *C. pipiens* and *O. punctor* (*HW-DW* and *TW-DW* allometries; Table 2). Elevation values ln(*a*) from the OLS regressions differed significantly in all 3 species only in the *TW-W* allometry (Table 3).

**Table 2.** Parameter estimates (with 95% confidence intervals in parentheses) of the SMA regressions (Eq. 2b) of the intraspecific length–mass allometries for the 3 mosquito species. Symbols: ln(*a*) = elevation, *b* = allometric slope, *SF *= Duan’s smearing factor, *R*^2^ = adjusted coefficient of determination, *N* = number of data points. Different superscripts indicate significant differences (adjusted *P* < 0.05) between species for the parameter of a given allometry

Allometry	Species	ln(*a*)	*b*	*SF*	*R* ^2^	*N*
Total body length *TL—*wet mass ***W***	*A. albopictus*	−11.00 (−11.20, −10.80) ^a^	3.00 (2.95, 3.06) ^a^	1.05	0.95	420
	*C. pipiens*	−10.80 (−10.90, −10.60) ^a^	2.94 (2.90, 2.99) ^a^	1.00	0.95	542
	*O. punctor*	−11.80 (−12.00, −11.50) ^b^	3.20 (3.14, 3.27) ^b^	1.02	0.93	351
Total body length *TL*—dry mass *DW*	*A. albopictus*	−11.20 (−11.60, −10.90) ^a^	2.82 (2.73, 2.91) ^a^	1.01	0.93	199
	*C. pipiens*	−13.50 (−13.60, −13.10) ^b^	3.39 (3.30, 3.48) ^b^	1.02	0.90	449
	*O. punctor*	−14.60 (−15.60, −13.80) ^c^	3.97 (3.64, 4.34) ^c^	1.02	0.66	171
Head width *HW*—Wet mass *W*	*A. albopictus*	−10.00 (−10.30, −9.70) ^a^	3.48 (3.37, 3.59) ^a^	1.01	0.90	420
	*C. pipiens*	−8.85 (−9.05, −8.64) ^b^	2.95 (2.88, 3.03) ^b^	1.03	0.89	542
	*O. punctor*	−10.10 (−10.50, −9.80) ^a^	3.45 (3.35, 3.56) ^a^	1.01	0.89	351
Head width *HW*—Dry mass *DW*	*A. albopictus*	−10.00 (−10.40, −9.71) ^a^	3.17 (3.05, 3.29) ^a^	1.01	0.90	199
	*C. pipiens*	−11.70 (−12.20, −11.3) ^b^	3.57 (3.42, 3.73) ^b^	1.07	0.80	449
	*O. punctor*	−12.10 (−12.90, −11.20) ^b^	3.73 (3.46, 4.02) ^b^	1.02	0.63	171
Thorax width *TW*—Wet mass *W*	*A. albopictus*	−9.30 (−9.45, −9.06) ^a^	3.15 (3.08, 3.22) ^a^	1.01	0.94	420
	*C. pipiens*	−8.10 (−8.21, −7.99) ^b^	2.65 (2.61, 2.68) ^b^	1.00	0.95	542
	*O. punctor*	−8.77 (−8.93, −8.61) ^c^	2.88 (2.83, 2.93) ^c^	1.00	0.96	351
Thorax width *TW*—Dry mass *DW*	*A. albopictus*	−9.26 (−9.50, −9.03) ^a^	2.83 (2.74, 2.91) ^a^	1.01	0.94	199
	*C. pipiens*	−10.50 (−10.70, −10.30) ^b^	3.09 (3.00, 3.17) ^b^	1.02	0.91	449
	*O. punctor*	−10.80 (−11.40, −10.10) ^b^	3.52 (3.24, 3.83)	1.01	0.69	171

**Table 3.** Parameter estimates (with 95% confidence intervals in parentheses) of the OLS regressions (Eq. 2b) of the intraspecific length–mass allometries for the 3 mosquito species. Traits (total body length, head width, thorax width) as predictors, and the masses as responses. Symbols: ln(*a*) = elevation, *b* = allometric slope, *SF* = Duan’s smearing factor, *R*^2^ = adjusted coefficient of determination, *N* = number of data points. Different superscripts indicate significant differences (adjusted *P* < 0.05) between species for the parameter of a given allometry

Allometry	Species	ln(*a*)	*b*	*SF*	*R* ^2^	*N*
Total body length *TL—*Wet mass *W*	*A. albopictus*	−10.84 (−11.00, −10.60) ^a^	2.96 (2.90, 3.02) ^a^	1.07	0.95	420
	*C. pipiens*	−10.60 (−10.80, −10.50) ^a^	2.90 (2.85, 2.94) ^a^	1.05	0.95	542
	*O. punctor*	−11.60 (−11.80, −11.30) ^b^	3.15 (3.08, 3.21) ^b^	1.03	0.93	351
Total body length *TL*—Dry mass *DW*	*A. albopictus*	−11.00 (−11.30, −10.70) ^a^	2.75 (2.66, 2.84) ^a^	1.08	0.93	199
	*C. pipiens*	−13.00 (−13.30, −12.70) ^b^	3.25 (3.16, 3.34) ^b^	1.13	0.90	449
	*O. punctor*	−12.70 (−13.70, −11.70) ^b^	3.16 (2.89, 3.43) ^b^	1.21	0.66	171
Head width *HW*—Wet mass *W*	*A. albopictus*	−9.63 (−9.93, −9.34) ^a^	3.33 (3.22, 3.44) ^a^	1.13	0.90	420
	*C. pipiens*	−8.48 (−8.69, −8.27) ^b^	2.82 (2.75, 2.90) ^b^	1.13	0.89	542
	*O. punctor*	−9.60 (−9.93, −9.28) ^a^	3.28 (3.17, 3.39) ^a^	1.05	0.89	351
Head width *HW*—Dry mass *DW*	*A. albopictus*	−9.70 (−10.00, −9.37) ^a^	3.05 (2.93, 3.17) ^a^	1.12	0.90	199
	*C. pipiens*	−10.80 (−11.20, −10.30) ^b^	3.23 (3.08, 3.39) ^a^	1.30	0.80	449
	*O. punctor*	−10.40 (−11.20, −9.60) ^ab^	3.18 (2.90, 3.46) ^a^	1.22	0.63	171
Thorax width *TW*—Wet mass *W*	*A. albopictus*	−9.07 (−9.26, −8.87) ^a^	3.08 (3.01, 3.15) ^a^	1.07	0.94	420
	*C. pipiens*	−8.00 (−8.10, −7.89) ^b^	2.61 (2.57, 2.65) ^b^	1.05	0.95	542
	*O. punctor*	−8.66 (−8.82, −8.49) ^c^	2.84 (2.79, 2.89) ^c^	1.02	0.89	351
Thorax width *TW*—Dry mass *DW*	*A. albopictus*	−9.11 (−9.35, −8.88) ^a^	2.77 (2.69, 2.86) ^a^	1.08	0.94	199
	*C. pipiens*	−10.20 (−10.40, −9.92) ^b^	2.97 (2.89, 3.05) ^b^	1.12	0.91	449
	*O. punctor*	−9.70 (−10.40, −9.10) ^ab^	2.81 (2.61, 3.02) ^ab^	1.18	0.69	171

Species-specific allometric slope estimates varied between 2.61 (*TW-W* allometry for *C. pipiens*) and 3.33 (*HW-W* allometry for *A. albopictus*) for OLS and between 2.65 (*TW-W* allometry for *C. pipiens*) and 3.97 (*TL-DW* allometry for *O. punctor*) for SMA depending on the predictor and response traits, but the 95% confidence intervals of most estimates overlapped or approached 3, indicating near-isometric scaling (Tables 2 and 3). The adjusted coefficients of determination of the SMA regressions were high (*R*^2^ ≥ 0.90) except for the allometries for the dry mass of *O. punctor* (*R*^2^ = 0.66–0.69). Thorax width and total body length were both markedly better predictors than head width. Thorax width was a good or slightly better predictor of individual mass than total body length, based on the *R*^2^ values of the SMA regressions (Table 2). Whether thorax width or body length gave a better fit (i.e., a higher *R*^2^ value) depended on the measure of body mass and species, making it impossible to identify the overall “better” linear trait in the OLS regression (Table 3).

SMA and OLS fit yielded similar *R*^2^ values (SMA: range = 0.63–0.96, mean ± SD = 0.87 ± 0.01; OLS: range 0.63–0.95, mean ± SD = 0.87 ± 0.01; Tables 2 and 3), but the OLS slope estimates were systematically lower than the SMA estimates (mean difference 7%, range 1.6%–21.0%). Finally, SMA inherently corrected for the underestimation of small individual mass, with Duan’s smearing factor values *SF* close to or equal to 1 (Table 2). Therefore, we focused only on SMA regressions.

**Fig. 2:**
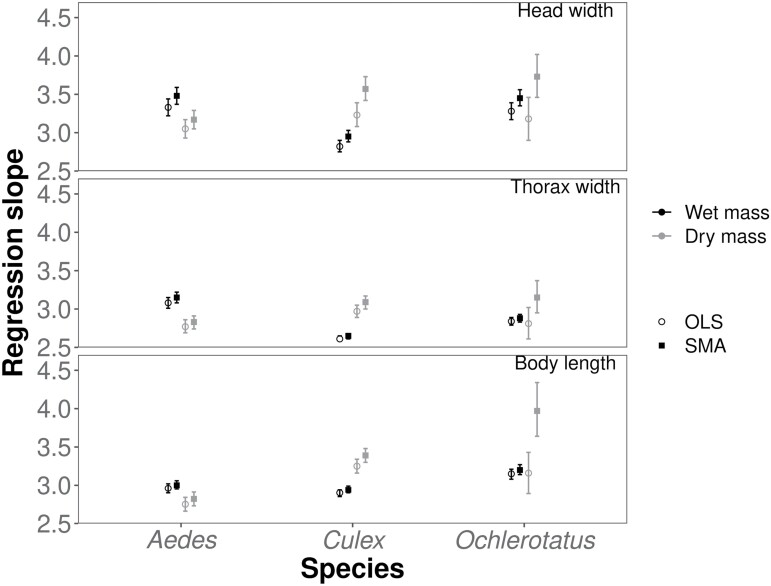
Estimates of the allometric slopes of the 3 mosquito species based on the 3 linear morphological traits and wet mass (black) and dry mass (gray) using OLS (open circles) and SMA (plain squares) regression. Error bars = 95% confidence intervals.Estimates of the allometric slopes of the 3 mosquito species *Aedes albopictus, Culex pipiens* and *Ochlerotatus punctor* based on the 3 linear morphological traits and wet mass and dry mass using OLS and SMA regression.

Using SMA, none of the relationships was characterized by a common allometric slope for all 3 species, although some slopes were not significantly different for 2 of the 3 species. That is, we found a similar slope of the body length*–*wet mass and head width*–*dry mass allometries in *A. albopictus* and *C. pipiens*, while the slopes were steeper in *O. punctor* (Fig. 2 and Table 2), and a similar slope of the head width*–*wet mass *A. albopictus* and *O. punctor*, while the slope was shallower in *C. pipiens* (Fig. 2 and Table 2). Slopes of the allometries linking thorax width to wet and dry mass and body length to dry mass were species-specific. They were lowest in *A. albopictus* and highest in *O. punctor* when considering dry mass and reversed when considering wet mass (Fig. 2 and Table 2). Allometric slopes for the 3 linear traits were higher for wet mass than for dry mass in *C. pipiens* and *O. punctor*, whereas the relationship was reversed in *A. albopictus* (Table 2).

### Length–Mass Allometries of Aquatic Dipterans

In addition to our data, we found 37 papers reporting a total of 218 values of length–mass allometric slopes for aquatic dipterans, including 98 slopes at the species level and 120 slopes at the genus (*N* = 58), (sub)family (*N* = 58) or whole order level (*N *= 5; Supplementary Table S8). Chironomidae was the most covered family (*N* = 108), followed by Simuliidae (*N* = 41) and Ceratopogonidae (*N* = 9). Sample sizes ranged widely between 6 and 746 individual data points (mean = 110, median = 38).

Most of the previously published length–mass relationships were based on dry mass (*N* = 202) rather than wet mass (*N* = 16) and used total body length (*N* = 138), head width (*N* = 52), interocular width (*N* = 9) or thorax width (*N* = 6) as the predictor. Preservation methods were more evenly spread among fresh weight (no preservation, *N* = 64), freezing (*N* = 31), and storage in ethanol (*N* = 31) or formaldehyde (*N* = 60).

**Fig. 3:**
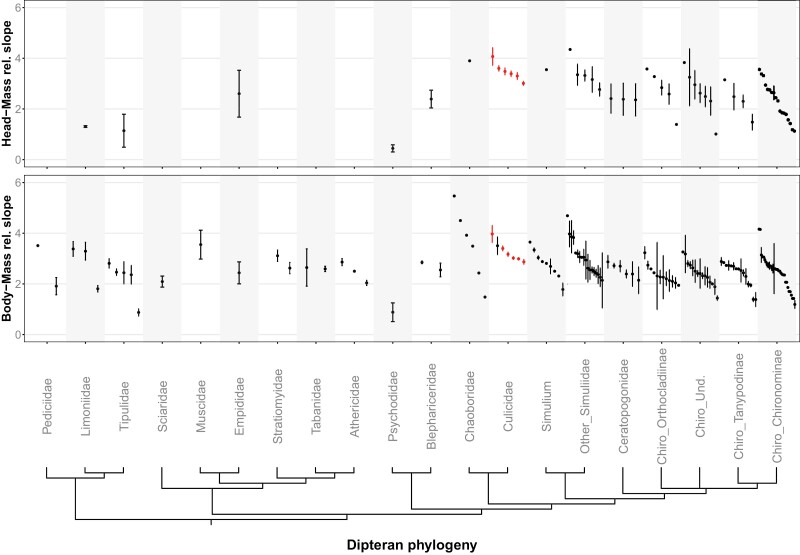
Dipteran phylogeny (left column) linked to published slopes of trait–mass relationships based on total body length (middle column) and head width (right column). Each unique combination of taxon and study is shown as a separate data point (our data in red). Points = mean estimates, horizontal bars = 95% confidence intervals (missing when the confidence interval is not reported or very small). Data are presented at the family level, except Chironomidae and Simuliidae plotted at a higher taxonomic resolution for clarity. See Supplementary Table S1 for details. Dipteran phylogeny linked to published slopes of trait–mass relationships based on total body length and head width. See Supplementary Table S1 for details

None of the analyses used SMA and instead used only linear regression of the log-transformed data (*N* = 80) or nonlinear least square regression of the raw data (*N* = 138). Allometric slope values ranged from 0.87 to 6.02 (mean ± SE = 2.64 ± 0.75) for total body length and from 0.44 to 4.35 (mean ± SE = 2.58 ± 0.90) for head width as the predictor, with substantial variation both within and between families (Fig. 3). We did not detect a significant phylogenetic signal in the body length–mass slopes (Abouheif’s test: Moran’s *I* = −0.05, *P* = 0.73) or in the head width–mass slopes (Abouheif’s test: Moran’s *I* = −0.13, *P* = 0.85).

**Fig. 4:**
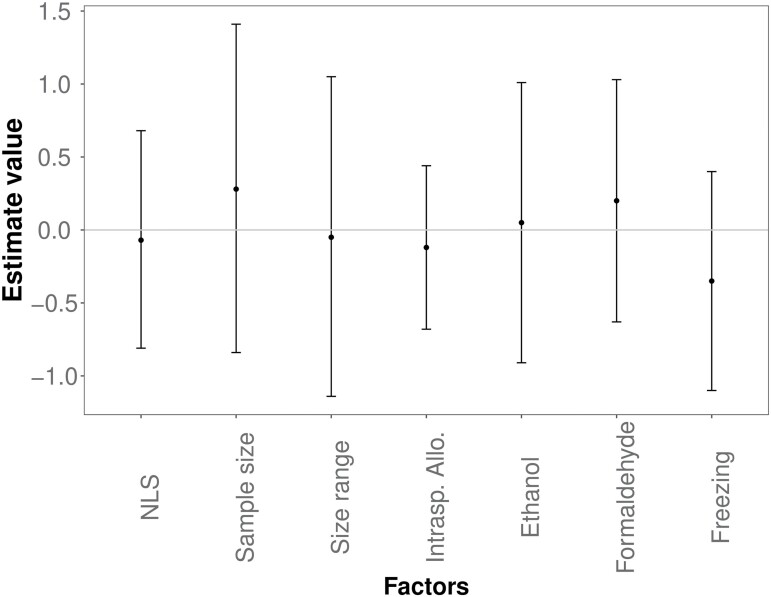
Estimates with a confidence interval of the most parsimonious GLMM of the allometric slope. The reference factor levels are linear regression for nonlinear regression (abbreviated as “NLS”), interspecific allometry for intraspecific allometry (abbreviated as “Intrasp. Allo.”), and fresh preservation method for freezing, respectively. Estimates with confidence intervals of the most parsimonious GLMM of the allometric slope.

**Fig. 5:**
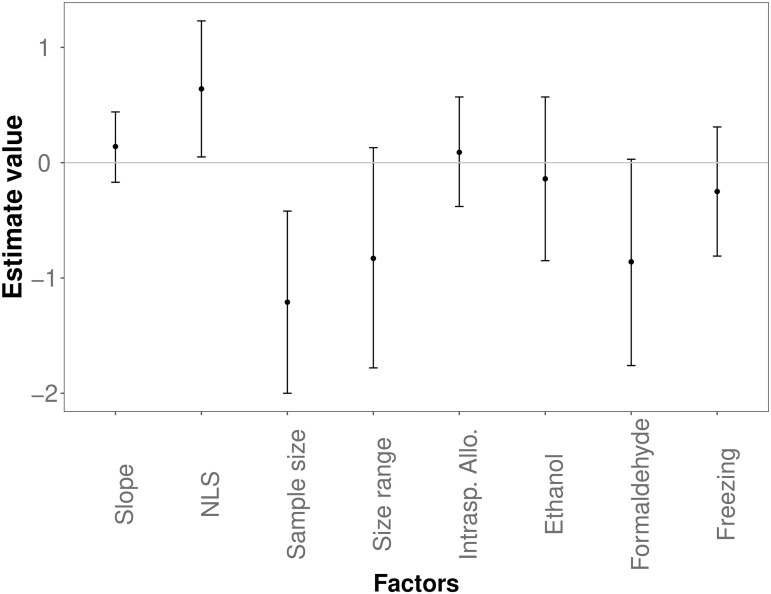
Estimates with a confidence interval of the most parsimonious GLMM of the 95% confidence interval of the allometric slopes. The reference factor levels are linear regression for nonlinear regression (abbreviated as “NLS”), interspecific allometry for intraspecific allometry (abbreviated as “Intrasp. Allo.”), and fresh preservation method for freezing, respectively. Estimates with confidence intervals of the most parsimonious GLMM of the 95% confidence interval of the allometric slopes.

The model, including the family, provided a better fit for the slope value but not for its 95% confidence interval (Supplementary Table S3). Model selection did not support the inclusion of ecological traits as relevant predictors of the allometric slope (Supplementary Tables S4 and S5) and its confidence interval (Supplementary Tables S6 and S7). Moreover, published allometric slope estimates were not affected by the estimation method (nonlinear least squares vs. OLS), sample size, size range, type of allometry, and preservation method (Fig. 4). However, wider size range and especially the larger sample size decreased the width of the 95% confidence interval (Fig. 5). Confidence interval was also wider for the estimates based on nonlinear least squares as compared to OLS, possibly related to the use of transformed vs. untransformed data (Fig. 5). Finally, the use of formaldehyde also tended to reduce the width of the confidence interval compared to all other methods used to process the samples.

## Discussion

We provide a detailed overview of existing data on intra- and interspecific length–mass allometries in the larvae of aquatic dipterans, including novel data on the larvae of 3 mosquito species. Our results show that the intraspecific length–mass allometries in mosquito larvae are comparable to those of other dipterans, but our conclusions are limited by the paucity of allometric studies of this family.

### Comparison of the Allometric Slopes of Larval Mosquitoes and Other Aquatic Dipterans

Our review of published allometries reveals that the current knowledge of length–mass allometries in most aquatic dipterans is patchy except for the families of Chironomidae and, to some extent, Simuliidae. Overall, the reported slopes for aquatic dipterans vary substantially, even within a family, subfamily, or genus. The observed lack of phylogenetic signal in the allometric slopes suggests that phylogeny does not control the length–mass relationship. It was already hypothesized that phylogeny puts weaker constraints on this relationship than environmental factors (Nylin and Gotthard 1998, Whitman and Agrawal 2009) since the length–mass relationships of aquatic insects from different geographical locations or with similar body shapes in different studies may yield strikingly different predictions, even for closely related taxa (Johnston and Cunjak 1999).

Despite extensive research on the Culicidae motivated by their ecological and public health importance, length–mass relationships in this family are almost unknown. In our study, the OLS- and SMA-based slopes of the most commonly used allometric relationships between body length and dry mass in *A. albopictus*, *C. pipiens*, and *O. punctor* were close to the value of 3 that corresponds to isometric growth. The only work on mosquitoes reports an OLS-based slope of 3.51 ± 0.35 (mean ± SE) for the body length—dry mass allometry of *Aedes* sp., using fresh individuals between 5.2 and 8.8 mm long (Oliphant and Hyslop 2020), compared to our estimates of 2.75 ± 0.05 (OLS regression) and 2.82 ± 0.05 (SMA regression) based on fresh individuals between 0.6 and 6.3 mm long. The different size ranges of measured individuals may explain the discrepancy between the OLS-based estimates. In addition, published estimates of the OLS-based slopes for dry mass include 2.43 and 4.50 for Chaoboridae (López et al. 1997, Johnston and Cunjak 1999), a sister group to the Culicidae (Wiegmann and Yeates 2017), and between 1.38 (Eklöf et al. 2017) and 4.16 (Fisher and Gray 1983) for Chironomidae (unweighted average of 2.40; Supplementary Table S1). That is, the slopes of length–mass relationships in larval mosquitoes found in our study are similar to the slopes reported for other dipteran families.

Of the 18 SMA slopes estimated across all combinations of traits in the 3 mosquito species, 5 and 10 were, respectively, lower and higher than the value corresponding to isometric growth (i.e., their 95% confidence intervals of the estimate did not include *b* = 3). This indicates the absence of a common pattern of ontogenetic change in the morphology of these species. Length–mass allometries of larvae of *O. punctor*, the largest of the 3 mosquito species examined in our study, were almost always steeper than those of *C. pipiens* and *A. albopictus*, highlighting a greater mass gain with total body size than in the latter 2 species. The marked differences in SMA-based allometric slopes based on thorax width among the 3 species suggest different growth patterns, which may reflect species-specific constraints on larval traits and trade-offs between larval and adult morphological traits, e.g., the need to minimize drag in larvae to facilitate escape movements against predators and to maximize the volume of flight muscles in adults (Helm et al. 2021).

Reliable data on individual body mass are essential for many types of ecological studies, especially for the calculation of secondary biomass and production (Banse and Mosher 1980, Eklöf et al. 2017). The lack of studies on length–mass relationship in larval Culicidae limits the ability to estimate their body mass, forcing authors to rely on other data. If the allometry of a taxon is missing, one can use the allometry of another closely related taxon or a higher taxonomic level (e.g., Reynolds and Benke 2005, Dekanová et al. 2022). Our study, which is one of the few focusing on mosquitoes, shows that their length–mass allometric slopes are within the range observed in other aquatic dipteran larvae. However, this range proved to be very large, sometimes even within the same family. Our results, therefore, emphasize the need to measure specific length–mass allometries directly whenever possible.

### Body Mass Estimates in Mosquito Larvae: Differences Between Morphological Traits

Overall, we found that the allometric slopes in larval mosquitoes vary considerably between species, and their values depend strongly on the choice of the linear trait and measure of body mass. Body mass of aquatic arthropods has been linked to various linear traits, including total body length (e.g., Benke et al. 1999, Miserendino 2001, Mährlein et al. 2016), head width or interocular width (Towers et al. 1994, Burgherr and Meyer 1997, Johnston and Cunjak 1999), femur length (Studier et al. 2002), forewing length (Mazón et al. 2020), and body width (Sohlström et al. 2018). These traits are not always equally suitable (Wenzel et al. 1990): the trait should be clearly delimited, correlate tightly with body mass, and preferably be planar to limit the measurement error when converting from a 3-dimensional structure to a 2-dimensional image (Cardini 2014). However, despite different accuracy in estimating the mass in length–mass relationships (e.g., Becker et al. 2009), studies that deal with a comparison of morphological traits to each other are quite rare, and thus, our study brings additional insight into this field.

Head width is a reliable predictor of larval stages (Shinkarenko et al. 1986) and could be used to estimate body mass of otherwise damaged individuals. However, because of heavy sclerotization, head width changes little or not at all within stages (Johnston and Cunjak 1999) and does not mark changes in body mass within a larval stage (Dyar 1890). This morphological trait disadvantage was also proved in our study since head width provided the least reliable estimation of body mass in all 3 mosquito species. Length–mass relationships in mosquito larvae should thus be based on more flexible body dimensions as previously recommended (e.g., Smock 1980, Towers et al. 1994, Burgherr and Meyer 1997). We found that thorax width is an equally good or better predictor of body mass than total body length in larval mosquitoes. We argue that thorax width is easier to measure, more clearly defined, and less prone to deformations, making it an ideal trait to estimate body mass in this group.

### Body Mass Estimates in Aquatic Dipterans: Differences Between Taxa, Traits, and Methods

Various factors can affect the estimated value of the slope of a length–mass relationship and its precision. In our study, precision improved with a larger sample size and size range. For this reason, we recommend using as many individuals as possible and including all instars for intraspecific allometries.

The allometric slopes obtained with the SMA regression were slightly higher than those obtained with the commonly used but conceptually less suitable OLS regression. Our analysis showed that the SMA regression does not require the correction for small individual size during the back-transformation, and SMA should, therefore, become the standard method for quantifying length–mass allometries (Warton and Weber 2002) also in dipterans.

We also observed substantial variation in the OLS-based slopes between morphological traits and species, both within and between families. This limits the possibility of deriving a single, universally applicable formula that could be used to estimate body mass of larval mosquitoes and other aquatic dipterans from a single linear dimension. Furthermore, most published length–mass relationships are based on preserved specimens. However, chemical preservation with ethanol or formaldehyde represents an important factor of precision of the allometry estimate since it can alter the wet mass (Landahl and Nagell 1978) and body length (Dermott and Paterson 1974, Lasenby et al. 1994) of the individuals, with idiosyncratic and often species-specific effects (Stanford 1973, Burgherr and Meyer 1997, Mährlein et al. 2016). Interestingly, slope estimates seem to vary less when individuals are preserved in formaldehyde, as observed in mayflies (Dekanová et al. 2023), but we are not aware of any direct comparison of the effect of this method to other processing options in aquatic dipterans.

Results of the analysis, including all aquatic dipterans, also imply that the allometric slopes are not influenced by ecological traits (i.e., current preference and locomotion type in our study) and the methods used to collect the data. However, the scope of our analyses of the role of ecological traits in length–weight relationships in aquatic dipterans was limited by the lack of data on other potentially relevant functional traits. Further studies on neglected taxa or targeting specific mechanisms, improving and standardizing the methods used for deriving length–mass relationships from raw data, are also needed to elucidate the effects of trade-offs and morphological constraints in the length–mass allometries of mosquitoes. Filling these gaps would help improve our understanding of variation in length–mass relationships in this ecologically and medically important group.

## Supplementary Material

ieae012_suppl_Supplementary_Material

## Data Availability

All experimental data are available from the Dryad Digital Repository https://doi.org/10.5061/dryad.j3tx95xn9.
