# Supplementary material for: Length–mass allometries of the larvae of aquatic dipterans: differences between taxa, morphological traits, and methods
Source: J Insect Sci. 2024 Feb 17;24(1):10. doi: 10.1093/jisesa/ieae012 (PMC10874217; doi:10.1093/jisesa/ieae012)
Supplement: ieae012_suppl_Supplementary_Material [file ieae012_suppl_supplementary_material.zip › MassLengthReg_Supp_Resubmission.docx]

Supplementary material for

**Length-mass allometries of the larvae of aquatic dipterans: differences between taxa, morphological traits and methods**

**Fig. S1.** Phylogenetic tree of aquatic Diptera. Only taxa with published trait-mass relationships (see Supplementary Table S8) and used in Abouheif’s test are included.

**Table S1.** Mean ± SE values of the different mass and linear dimension traits of the four larval stages of *A. albopictus*, *C. pipiens* and *O. punctor*.

**Table S2.** Parameter values of log_10_-transformed morphological trait-mass linear regressions for *A. albopictus*, *C. pipiens* and *O. punctor*.

**Table S3.** Summary of GLMMs of allometric slope and slope deviance including Family as additional predictor.

**Table S4**. Comparison of GLMMs of allometric slope including ecological traits as additional predictors.

**Table S5.** Summary of GLMMs of allometric slope including ecological traits as additional predictors.

**Table S6**. Comparison of GLMMs of 95% confidence interval of allometric slope including ecological factors as additional predictors.

**Table S7.** Summary of GLMMs of 95% confidence intervals of allometric slope including ecological traits as additional predictors.

**Table S8**. Overview of previously published studies of length-mass relationships in aquatic Diptera.

**Fig. S1.** Phylogenetic tree of aquatic Diptera based on Wiegmann et al. (2011), Lifemap (de Vienne 2016), and for specific taxonomic groups, Cranston et al. (2012), Krosch et al. (2017), and Krosch et al. (2022) for Chironomidae; Becker et al. (2010) for Culicidae; Ribeiro (2008) for Tipulomorpha; Evans and Adler (2000), Gil-Azevedo and Coscarón (2020); LaRue et al. (2009) for Simuliidae. Only taxa with published trait-mass relationships (see Table S8) and used in Abouheif’s test are included.


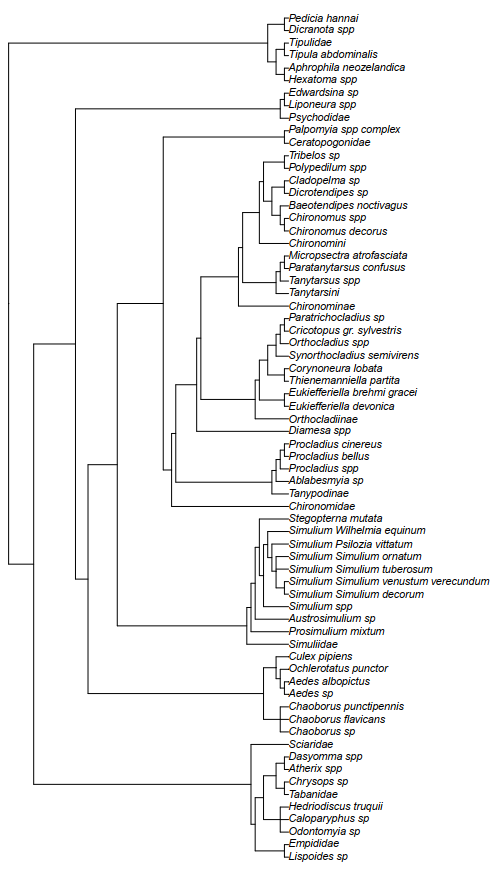


**Table S1:** Mean ± SE values of the different mass and linear dimension traits (wet mass (*W*, mg) and dry mass (*DW*, mg), total body length (*TL*, mm), head width (*HW*, mm) and thorax width (*TL*, mm)) of the four larval stages (L1-L4) of the three mosquito species. Different superscripts indicate significant differences in a given trait among all 12 combinations of species and instars (GLM and LS means test, adjusted *p* < 0.05).

| **Size measure** | **Species** | **L1** | **L2** | **L3** | **L4** |
| --- | --- | --- | --- | --- | --- |
| *W* (10^-4^ g) | *Aedes albopictus* | 0.21 ± 0.10 ^a^ | 1.49 ± 0.96 ^c^ | 6.63 ± 3.44 ^d^ | 13.4 ± 5.21 ^g^ |
|  | *Culex pipiens* | 0.33 ± 0.17 ^b^ | 1.08 ± 0.46 ^c^ | 2.99 ± 1.35 ^e^ | 14.7 ± 6.18 ^g^ |
|  | *Ochlerotatus punctor* | 1.40 ± 0.19 ^ce^ | 6.77 ± 2.59 ^d^ | 22.60 ± 8.35 ^f^ | 50.2 ± 16.5 ^h^ |
| *DW* (10^-4^ g) | *Aedes albopictus* | 0.04 ± 0.02 ^a^ | 0.24 ± 0.11 ^b^ | 0.77 ± 0.27 ^e^ | 1.81 ± 0.70 ^f^ |
|  | *Culex pipiens* | 0.03 ± 0.02 ^a^ | 0.09 ± 0.07 ^c^ | 0.22 ± 0.16 ^b^ | 1.60 ± 1.06 ^f^ |
|  | *Ochlerotatus punctor* | NA | 0.60 ± 0.39 ^d^ | 2.05 ± 0.98 ^f^ | 4.18 ± 1.81 ^g^ |
| *TL* (mm) | *Aedes albopictus* | 1.21 ± 0.26 ^a^ | 2.36 ± 0.43 ^c^ | 3.89 ± 0.68 ^e^ | 4.94 ± 0.62 ^g^ |
|  | *Culex pipiens* | 1.42 ± 0.22 ^b^ | 2.08 ± 0.29 ^c^ | 2.96 ± 0.35 ^d^ | 5.21 ± 0.71 ^g^ |
|  | *Ochlerotatus punctor* | 2.52 ± 0.20 ^cd^ | 4.19 ± 0.52 ^e^ | 5.86 ± 0.65 ^f^ | 7.80 ± 0.94 ^h^ |
| *HW* (mm) | *Aedes albopictus* | 0.24 ± 0.03 ^a^ | 0.47 ± 0.032 ^d^ | 0.66 ± 0.06 ^f^ | 0.82 ± 0.05 ^i^ |
|  | *Culex pipiens* | 0.28 ± 0.01 ^b^ | 0.46 ± 0.026 ^c^ | 0.70 ± 0.04 ^g^ | 1.07 ± 0.08 ^h^ |
|  | *Ochlerotatus punctor* | 0.46 ± 0.03 ^c^ | 0.74 ± 0.038 ^e^ | 1.06 ± 0.06 ^h^ | 1.39 ± 0.13 ^j^ |
| *TW* (mm) | *Aedes albopictus* | 0.25 ± 0.05 ^a^ | 0.48 ± 0.10 ^c^ | 0.75± 0.13 ^e^ | 0.93 ± 0.14 ^d^ |
|  | *Culex pipiens* | 0.31 ± 0.06 ^b^ | 0.49 ± 0.08 ^c^ | 0.71 ± 0.10 ^e^ | 1.31 ± 0.21 ^g^ |
|  | *Ochlerotatus punctor* | 0.56 ± 0.08 ^c^ | 0.97 ± 0.14 ^d^ | 1.45 ± 0.17 ^f^ | 1.96 ± 0.20 ^h^ |

**Table S2:** Linear regression (ordinary least squares, OLS) linking log-transformed morphological traits (head width or body length) as predictor and (dry or wet) mass as response for the three mosquito species. Parameters: ln(*a*) = intercept; *b* = slope coefficient; *SF* = Duan’s smearing factor, *R*^2^ = adjusted coefficients of determination. Different superscripts indicate significant differences (adjusted *p* < 0.05) between species for the given parameter of a given allometry.

| **Allometry** | **Species** | **ln(*a*)** | ***b*** | ***SF*** | ***R*^2^** |
| --- | --- | --- | --- | --- | --- |
| Total body length *TL* - Wet mass *W* | *A. albopictus* | -1.96 (-1.97, -1.95) ^a^ | 2.94 (2.92, 2.96) ^a^ | 1.07 | 0.95 |
|  | *C. pipiens* | -1.94 (-1.95, -1.93) ^a^ | 2.91 (2.88, 2.94) ^a^ | 1.05 | 0.95 |
|  | *O. punctor* | -2.05 (-2.08, -2.02) ^b^ | 3.07 (3.02, 3.12) ^b^ | 1.03 | 0.93 |
| Total body length *TL* - Dry mass *DW* | *A. albopictus* | -2.77 (-2.8, -2.74) ^a^ | 2.77 (2.72, 2.82) ^a^ | 1.08 | 0.94 |
|  | *C. pipiens* | -3.21 (-3.24, -3.18) ^b^ | 3.23 (3.18, 3.28) ^b^ | 1.13 | 0.9 |
|  | *O. punctor* | -3.33 (-3.46, -3.2) ^b^ | 3.23 (3.05, 3.41) ^b^ | 1.21 | 0.66 |
| Head width *HW* - Wet mass *W* | *A. albopictus* | 0.38 (0.36, 0.4) ^a^ | 3.41 (3.36, 3.46) ^a^ | 1.13 | 0.90 |
|  | *C. pipiens* | -0.02 (-0.03, -0.01) ^b^ | 2.84 (2.8, 2.88) ^b^ | 1.13 | 0.89 |
|  | *O. punctor* | 0.23 (0.2, 0.26) ^c^ | 3.2 (3.19, 3.21) ^c^ | 1.05 | 0.89 |
| Head width *HW* - Dry mass *DW* | *A. albopictus* | -0.55 (-0.58, -0.52) ^a^ | 3.12 (3.05, 3.19) ^a^ | 1.12 | 0.9 |
|  | *C. pipiens* | -1.08 (-1.1, -1.06) ^b^ | 3.11 (3.04, 3.18) ^a^ | 1.3 | 0.8 |
|  | *O. punctor* | -0.9 (-1.1, -0.7) ^c^ | 3.23 (3.04, 3.42) ^a^ | 1.22 | 0.63 |
| Thorax width *TW* - Wet mass *W* | *A. albopictus* | 0.17 (0.16, 0.18) ^a^ | 3.1 (3.07, 3.13) ^a^ | 1.07 | 0.94 |
|  | *C. pipiens* | -0.17 (-0.16, -0.18) ^b^ | 2.61 (2.59, 2.63) ^b^ | 1.05 | 0.95 |
|  | *O. punctor* | -0.13 (-0.14, -0.12) ^c^ | 2.81 (2.76, 2.86) ^c^ | 1.02 | 0.89 |
| Thorax width *TW* - Dry mass *DW* | *A. albopictus* | -0.8 (-0.82, -0.78) ^a^ | 2.76 (2.71, 2.81) ^a^ | 1.08 | 0.94 |
|  | *C. pipiens* | -1.25 (-1.26, -1.24) ^b^ | 2.93 (2.88, 2.98) ^b^ | 1.12 | 0.91 |
|  | *O. punctor* | -1.31 (-1.34, -1.26) ^c^ | 2.92 (2.77, 3.07) ^b^ | 1.18 | 0.69 |

**Table S3:** GLMMs of allometric slope and slope deviance including Family, fitting method, the standardized sample size and size range, the type of allometry and the preservation method as fixed factors, and the identity of the original paper as a random factor. See Material and Methods for detailed description of the predictors. Note that no slope deviance was reported for the Chaoboridae. The tested level of each predictor is indicated in square bracket. The reference factor levels are Linear regression for Fitting method, Interspecific allometry for Allometry and Fresh for Preservation method, and Culicidae for Family. Original source paper used as a random effect to account for possible lack of independence among the results of analyses of multiple datasets in the same paper

|  | **Allometric slope** | | | **Slope deviance** | | |
| --- | --- | --- | --- | --- | --- | --- |
| *Predictors* | *Estimate* | *CI* |  | *Estimate* | *CI* |  |
| (Intercept) | 3.63 | 2.66 – 4.60 |  | 0.73 | -0.19 – 1.66 |  |
| Fitting method [NLS] | 0.50 | -0.14 – 1.14 |  | 0.21 | -0.34 – 0.76 |  |
| Stdr Sample size | 0.45 | -0.35 – 1.25 |  | -0.94 | -1.43 to -0.46 |  |
| Stdr Size Range | -0.22 | -1.21 – 0.76 |  | -0.95 | -1.65 to -0.25 |  |
| Allometry [Intraspecific] | -0.33 | -0.83 – 0.17 |  | 0.02 | -0.35 – 0.39 |  |
| Preservation method [Ethanol] | 0.26 | -0.54 – 1.05 |  | -0.18 | -0.88 – 0.52 |  |
| Preservation method [Formaldehyde] | 0.42 | -0.32 – 1.17 |  | -0.30 | -1.03 – 0.43 |  |
| Preservation method [Freezing] | -0.33 | -1.04 – 0.39 |  | -0.10 | -0.73 – 0.54 |  |
| Family [Chironomidae] | -1.33 | -2.43 to -0.23 |  | 0.21 | -0.59 – 1.00 |  |
| Family [Athericidae] | -1.38 | -2.77 – 0.00 |  | -0.20 | -1.14 – 0.75 |  |
| Family [Blephariceridae] | -1.36 | -2.70 to -0.01 |  | -0.29 | -1.20 – 0.61 |  |
| Family [Ceratopogonidae] | -1.01 | -2.34 – 0.32 |  | 0.08 | -0.80 – 0.97 |  |
| Family [Chaoboridae] | -0.65 | -2.39 – 1.10 |  | - | - |  |
| Family [Limoniidae] | -1.12 | -2.35 – 0.12 |  | -0.15 | -0.98 – 0.68 |  |
| Family [Muscidae] | -0.33 | -2.10 – 1.44 |  | 0.35 | -0.71 – 1.41 |  |
| Family [Pediciidae] | -2.07 | -3.78 to -0.36 |  | 0.01 | -1.08 – 1.11 |  |
| Family [Simuliidae] | -0.96 | -2.10 – 0.18 |  | -0.10 | -0.92 – 0.73 |  |
| Family [Stratiomyidae] | 0.48 | -0.82 – 1.78 |  | -0.11 | -1.03 – 0.80 |  |
| Family [Tabanidae] | -0.26 | -1.97 – 1.46 |  | 0.72 | -0.33 – 1.76 |  |
| Family [Tipulidae] | -1.74 | -3.24 to -0.23 |  | 0.65 | -0.33 – 1.63 |  |
| Slope | - | - |  | 0.09 | -0.09 – 0.28 |  |
| **Random Effects** | | | | | | |
| σ^2^ | 0.42 | | | 0.11 | | |
| τ_00_ | 0.23 _Original.paper_ | | | 0.10 _Original.paper_ | | |
| ICC | 0.36 | | | 0.48 | | |
| *N*_random_ | 24 _Original.paper_ | | | 12 _Original.paper_ | | |
| Observations | 152 | | | 81 | | |
| Marginal R^2^ / Conditional R^2^ | 0.244 / 0.512 | | | 0.371 / 0.670 | | |

**Table S4**: Comparison of gaussian GLMMs of allometric slopes using the ecological traits (locomotion and current preference), fitting method, standardized sample size and size range, type of allometry and preservation method as fixed factors and the identity of the original paper as a random factor. Model structure: ‘Baseline’ structure includes the same predictors as the ‘Baseline’ model. df = degrees of freedom, ln(*L*) *=* log-likelihood, ΔAICc = difference of the corrected Akaike Information Criterion value from the most parsimonious model, *w* = Akaike weight.

| **Model name** | **Model structure (fixed effects)** | **df** | **ln(*L*)** | **ΔAICc** | ***w*** |
| --- | --- | --- | --- | --- | --- |
| Baseline | ~ Fitting method + stdr sample size + stdr size range + allometry type + preservation method | 10 | 0 | 0 | 0.956 |
| Locomotion | ~ Baseline + Locomotion | 13 | 0.7 | 6.4 | 0.038 |
| Current preference | ~ Baseline + Current preference | 14 | 0.2 | 10.2 | 0.006 |
| Full | ~ Baseline + Locomotion + Current preference | 17 | 0.6 | 17.8 | <0.001 |

**Table S5:** Gaussian GLMMs of allometric slopes using the ecological traits (locomotion and current preference), fitting method, standardized sample size and size range, type of allometry and preservation method as fixed factors and the identity of the original paper as a random factor. See Table S4 for model names. ΔAICc = difference of the corrected Akaike Information Criterion value from the most parsimonious model (i.e. the ‘Baseline’ model). See Material and Methods for the description of the explanatory variables. The tested level of each predictor is indicated in square bracket. The reference factor levels are Linear regression for Fitting method, Interspecific allometry for Allometry and Fresh for Preservation method, and Culicidae for Family. Original source paper used as a random effect to account for possible lack of independence among the results of analyses of multiple datasets in the same paper

| **Model** | **Baseline** | | **Locomotion** | | | **Current preference** | | | **Full** | | |
| --- | --- | --- | --- | --- | --- | --- | --- | --- | --- | --- | --- |
| *Predictors* | *Estimate* | *CI* | *Estimate* | *CI* |  | *Estimate* | *CI* |  | *Estimate* | *CI* |  |
| (Intercept) | 3.04 | 2.29 – 3.78 | 3.36 | 2.54 – 4.18 |  | 3.14 | 2.34 – 3.94 |  | 3.29 | 2.28 – 4.30 |  |
| Fitting method [NLS] | -0.07 | -0.81 – 0.68 | -0.00 | -0.73 – 0.73 |  | -0.01 | -0.82 – 0.81 |  | 0.08 | -0.93 – 1.10 |  |
| Stdr Sample size | 0.28 | -0.84 – 1.41 | 0.31 | -0.95 – 1.57 |  | 0.31 | -0.83 – 1.45 |  | 0.34 | -0.94 – 1.62 |  |
| Stdr Size Range | -0.05 | -1.14 – 1.05 | 0.02 | -1.05 – 1.08 |  | -0.10 | -1.32 – 1.11 |  | -0.26 | -1.54 – 1.01 |  |
| Allometry [Intraspecific] | -0.12 | -0.68 – 0.44 | -0.19 | -0.75 – 0.37 |  | -0.17 | -0.76 – 0.42 |  | -0.21 | -0.83 – 0.41 |  |
| PreservationMethod [Ethanol] | 0.05 | -0.91 – 1.01 | 0.23 | -0.65 – 1.10 |  | 0.16 | -0.86 – 1.17 |  | 0.28 | -0.94 – 1.49 |  |
| PreservationMethod [Formaldehyde] | 0.20 | -0.63 – 1.03 | 0.16 | -0.59 – 0.91 |  | 0.01 | -0.94 – 0.96 |  | 0.08 | -1.05 – 1.22 |  |
| PreservationMethod [Freezing] | -0.35 | -1.10 – 0.40 | -0.17 | -0.93 – 0.59 |  | -0.36 | -1.12 – 0.40 |  | -0.37 | -1.24 – 0.50 |  |
| Locomotion [Burrowing] | - | - | -0.57 | -1.39 – 0.24 |  | - | - |  | -0.04 | -1.27 – 1.19 |  |
| Locomotion [Sessile] | - | - | -0.46 | -1.25 – 0.34 |  | - | - |  | -0.34 | -1.51 – 0.83 |  |
| Locomotion [Sprawling] | - | - | -0.69 | -1.51 – 0.14 |  | - | - |  | -0.27 | -1.38 – 0.85 |  |
| CurrentPref [linear] | - | - | - | - |  | -0.58 | -1.58 – 0.42 |  | -0.37 | -1.73 – 0.99 |  |
| CurrentPref [quadratic] | - | - | - | - |  | 0.28 | -0.59 – 1.14 |  | 0.12 | -1.07 – 1.31 |  |
| CurrentPref [cubic] | - | - | - | - |  | -0.33 | -0.90 – 0.24 |  | -0.25 | -0.97 – 0.47 |  |
| CurrentPref [4th degree] | - | - | - | - |  | -0.17 | -0.58 – 0.24 |  | -0.25 | -0.70 – 0.21 |  |
| ΔAICc | 0 | | 6.4 | | | 10.2 | | | 17.8 | | |
| **Random Effects** | | | | | | | | | | | |
| σ^2^ | 0.45 | | 0.47 | | | 0.45 | | | 0.45 | | |
| τ_00_ | 0.23 _Original.paper_ | | 0.14 _Original.paper_ | | | 0.25 _Original.paper_ | | | 0.38 _Original.paper_ | | |
| ICC | 0.34 | | 0.23 | | | 0.36 | | | 0.46 | | |
| *N*_random_ | 16 _Original.paper_ | | 16 _Original.paper_ | | | 16 _Original.paper_ | | | 16 _Original.paper_ | | |
| Observations | 98 | | 98 | | | 98 | | | 98 | | |
| Marginal R^2^ / Conditional R^2^ | 0.025 / 0.354 | | 0.075 / 0.288 | | | 0.064 / 0.400 | | | 0.082 / 0.505 | | |

**Table S6**: Comparison of gaussian GLMMs of allometric slope 95% confidence interval using the ecological traits (current preference and locomotion type), fitting method, standardized sample size and size range, type of allometry, value of the allometric slope and preservation method as fixed factors and the identity of the original paper as random factor. Model structure: ‘Baseline’ structure includes the same predictors as the ‘Baseline’ model. df = degrees of freedom, ln(*L*) *=* log-likelihood, ΔAICc = difference of the corrected Akaike Information Criterion values compared to the most parsimonious model (i.e. the base model), *w* = Akaike weight.

| **Model name** | **Model structure (fixed effects)** | **df** | **ln(*L*)** | **ΔAICc** | ***w*** |
| --- | --- | --- | --- | --- | --- |
| Baseline | ~ Fitting method + strd sample size + strd size range + allometry type+ slope + preservation method | 11 | 1.4 | 0 | 1 |
| Locomotion | ~ Baseline + Locomotion | 14 | 1.3 | 14.8 | <0.001 |
| Current preference | ~ Baseline + Current preference | 14 | 0 | 17.4 | <0.001 |
| Full | ~ Baseline + Locomotion + Current preference | 17 | 0.7 | 35.2 | <0.001 |

**Table S7:** GLMMs of allometric slope 95% confidence interval as explained variable, using the fitting method, the standardized sample size and size range, the type of allometry, the allometric slope value and the preservation method, as tested fixed factors the current preference and the locomotion type as systematic fixed factors, and, the identity of the original paper as random factor. ΔAICc is the difference of the corrected Akaike Information Criterion values compared with the most parsimonious model. The tested level of each predictor is indicated in square bracket. The reference factor levels are Linear regression for Fitting method, Interspecific allometry for Allometry and Fresh for Preservation method, and Culicidae for Family. Original source paper used as a random effect to account for possible lack of independence among the results of analyses of multiple datasets in the same paper. See Material & Methods for the description of the explanatory variables.

| **Model** | **Baseline** | | | **Locomotion** | | | **Current preference** | | | **Full** | | |
| --- | --- | --- | --- | --- | --- | --- | --- | --- | --- | --- | --- | --- |
| *Predictors* | *Estimate* | *CI* | *p* | *Estimate* | *CI* | *p* | *Estimate* | *CI* | *p* | *Estimate* | *CI* | *p* |
| (Intercept) | 0.60 | -0.51 – 1.70 | 0.279 | 0.05 | -1.51 – 1.61 | 0.949 | 0.73 | -0.52 – 1.99 | 0.238 | -0.03 | -1.78 – 1.72 | 0.973 |
| Fitting method [NLS] | 0.64 | 0.05 – 1.23 | **0.035** | 0.61 | -0.26 – 1.47 | 0.161 | 0.62 | -0.31 – 1.55 | 0.180 | 0.80 | -0.41 – 2.01 | 0.181 |
| Stdr Sample size | -1.21 | -2.00 – -0.42 | **0.004** | -0.96 | -1.91 – -0.01 | **0.047** | -1.29 | -2.14 – -0.45 | **0.004** | -1.05 | -2.08 – -0.01 | **0.047** |
| Stdr Size Range | -0.83 | -1.78 – 0.13 | 0.088 | -0.72 | -1.79 – 0.36 | 0.181 | -0.85 | -1.97 – 0.26 | 0.128 | -0.67 | -1.89 – 0.56 | 0.270 |
| Allometry [Intraspecific] | 0.09 | -0.38 – 0.57 | 0.688 | -0.00 | -0.55 – 0.55 | 0.995 | 0.02 | -0.55 – 0.58 | 0.946 | -0.07 | -0.68 – 0.55 | 0.817 |
| PreservationMethod [Ethanol] | -0.14 | -0.85 – 0.57 | 0.692 | -0.07 | -1.22 – 1.07 | 0.894 | -0.07 | -1.14 – 1.00 | 0.896 | 0.06 | -1.34 – 1.47 | 0.926 |
| PreservationMethod [Formaldehyde] | -0.86 | -1.76 – 0.03 | 0.059 | -1.05 | -2.47 – 0.38 | 0.143 | -0.70 | -1.91 – 0.51 | 0.245 | -1.04 | -2.69 – 0.61 | 0.203 |
| PreservationMethod [Freezing] | -0.25 | -0.81 – 0.31 | 0.365 | -0.13 | -0.92 – 0.66 | 0.735 | -0.25 | -0.92 – 0.41 | 0.439 | -0.16 | -1.05 – 0.73 | 0.717 |
| Slope | 0.14 | -0.17 – 0.44 | 0.360 | 0.24 | -0.12 – 0.60 | 0.185 | 0.08 | -0.26 – 0.43 | 0.625 | 0.21 | -0.18 – 0.61 | 0.278 |
| Locomotion [Burrowing] | - | - | - | 0.13 | -0.71 – 0.97 | 0.755 | - | - | - | 0.18 | -0.85 – 1.22 | 0.720 |
| Locomotion [Sessile] | - | - | - | 0.48 | -0.39 – 1.34 | 0.267 | - | - | - | 0.63 | -0.44 – 1.70 | 0.233 |
| Locomotion [Sprawling] | - | - | - | 0.09 | -0.70 – 0.88 | 0.818 | - | - | - | 0.18 | -0.76 – 1.12 | 0.695 |
| CurrentPref [linear] | - | - | - | - | - | - | -0.21 | -0.92 – 0.49 | 0.538 | -0.33 | -1.24 – 0.58 | 0.453 |
| CurrentPref [quadratic] | - | - | - | - | - | - | -0.02 | -0.58 – 0.53 | 0.927 | 0.17 | -0.58 – 0.91 | 0.647 |
| CurrentPref [cubic] | - | - | - | - | - | - | -0.01 | -0.40 – 0.38 | 0.965 | 0.01 | -0.46 – 0.48 | 0.964 |
| ΔAICc |  | 0 |  |  | 14.8 |  |  | 17.4 |  |  | 35.2 |  |
| **Random Effects** | | | | | | | | | | | | |
| σ^2^ | 0.15 | | | 0.15 | | | 0.16 | | | 0.16 | | |
| τ_00_ | 0.04 _Original.paper_ | | | 0.16 _Original.paper_ | | | 0.12 _Original.paper_ | | | 0.22 _Original.paper_ | | |
| ICC | 0.23 | | | 0.51 | | | 0.43 | | | 0.58 | | |
| *N*_random_ | 8 _Original.paper_ | | | 8 _Original.paper_ | | | 8 _Original.paper_ | | | 8 _Original.paper_ | | |
| Observations | 37 | | | 37 | | | 37 | | | 37 | | |
| Marginal R^2^ / Conditional R^2^ | 0.405 / 0.540 | | | 0.353 / 0.686 | | | 0.329 / 0.616 | | | 0.318 / 0.716 | | |

**Table S8 (provided as a separate Excel file)**: Overview of published studies of length-mass relationships (Slope and standard error of the Slope, SE_Slope) in aquatic Diptera including the fitting method (NLS = nonlinear least-squares regression, LR = linear regression), fitted functional form (*M* = mass, *L* = morphological trait), type of mass (dry or wet), morphological traits used as predictors, parameter estimates (with standard error if available), coefficient of determination (*R*^2^), sample size (*N*), and size range (mm). The intercept *a* is reported untransformed when the allometry (*M* = *a* *L^b^*) was fitted directly and as ln(*a*) when the linearized relationship In(*M*) = In(*a*) + b In(*L*) was used. Some of the published relationships were extracted from a secondary source ((1) = Benke et al. 1999, (2) = Johnston and Cunjak 1999). Some papers provided multiple slope values for the same trait-mass relationship from different populations (e.g. Shadrin et al. 2019): each population were considered independently in our study.

**References included in Table S8.**

Allan JD. 1984. The Size Composition of Invertebrate Drift in a Rocky Mountain Stream. Oikos. 43(1):68. doi:10.2307/3544247.

Becker N, Petrić D, Zgomba M, Boase C, Madon M, Dahl C, Kaiser A. 2010. Mosquitoes and their control: Second edition. Berlin Heidelberg: Springer-Verlag Berlin Heidelberg.

Benke AC, Huryn AD, Smock LA, Wallace JB. 1999. Length-Mass Relationships for Freshwater Macroinvertebrates in North America with Particular Reference to the Southeastern United States. J North Am Benthol Soc. 18(3):308–343. doi:10.2307/1468447.

Burgherr P, Meyer EI. 1997. Regression analysis of linear body dimensions vs. dry mass in stream macroinvertebrates. Arch für Hydrobiol. 139(1):101–112.

Butler MG. 1982. Production dynamics of some arctic Chironomus larvae. Limnol Oceanogr. 27(4):728–736. doi:10.4319/lo.1982.27.4.0728.

Cranston PS, Hardy NB, Morse GE. 2012. A dated molecular phylogeny for the Chironomidae (Diptera). Syst Entomol. 37(1):172–188. doi:10.1111/j.1365-3113.2011.00603.x.

Dekanová V, Venarsky MP, Bunn SE. 2022. Length–mass relationships of Australian aquatic invertebrates. Austral Ecol. 47(1):120–126. doi:10.1111/aec.13077.

Dumont HJ, Balvay G. 1979. The dry weight estimate of Chaoborus flavicans (Meigen) as a function of length and instars. Hydrobiologia. 64(2):139–145. doi:10.1007/BF00023189.

Eaton KA. 1983. The life history and production of Chaoborus punctipennis (Diptera: Chaoboridae) in Lake Norman, North Carolina, U.S.A. Hydrobiologia. 106(3):247–252. doi:10.1007/BF00008123.

Eklöf J, Austin Å, Bergström U, Donadi S, Eriksson BDHK, Hansen J, Sundblad G. 2017. Size matters: relationships between body size and body mass of common coastal, aquatic invertebrates in the Baltic Sea. PeerJ. 5:e2906. doi:10.7717/peerj.2906.

Evans CL, Adler PH. 2000. Microsculpture and phylogenetic significance of the spermatheca of black flies (Diptera: Simuliidae). Can J Zool. 78(8):1468–1482. doi:10.1139/z00-078.

Fisher SG, Gray LJ. 1983. Secondary Production and Organic Matter Processing by Collector Macroinvetebrates in a Desert Stream. Ecology. 64(5):1217–1224. doi:10.2307/1937830.

Gil-Azevedo LH, Coscarón S. 2020. Comprehensive phylogeny of Simulium (Psilopelmia) Enderlein (Diptera: Simuliidae) – classification tested against comparative morphology. Arthropod Syst Phylogeny. 78(3):405–425. doi:10.26049/ASP78-3-2020-04.

Grzybkowska M. 1985. The growth of Procladius cinereus Goetghebuer. 1936 (Diptera. Chironomidae) larva. Acta Hydrobiol. 27:81–89.

Johnston TA, Cunjak RA. 1999. Dry mass–length relationships for benthic insects: a review with new data from Catamaran Brook, New Brunswick, Canada. Freshw Biol. 41(4):653–674. doi:10.1046/j.1365-2427.1999.00400.x.

Krosch MN, Cranston PS, Bryant LM, Strutt F, McCluen SR. 2017. Towards a dated molecular phylogeny of the Tanypodinae (Chironomidae, Diptera). Invertebr Syst. 31(3):302. doi:10.1071/IS16046.

Krosch MN, Silva FL, Ekrem T, Baker AM, Bryant LM, Stur E, Cranston PS. 2022. A new molecular phylogeny for the Tanypodinae (Diptera: Chironomidae) places the Australian diversity in a global context. Mol Phylogenet Evol. 166:107324. doi:10.1016/j.ympev.2021.107324.

Ladle M, Bass JAB, Jenkins WR. 1972. Studies on production and food consumption by the larval simuliidae (Diptera) of a chalk stream. Hydrobiologia. 39(3):429–448. doi:10.1007/BF00046654.

LaRue B, Gaudreau C, Bagre HO, Charpentier G. 2009. Generalized structure and evolution of ITS1 and ITS2 rDNA in black flies (Diptera: Simuliidae). Mol Phylogenet Evol. 53(3):749–757. doi:10.1016/j.ympev.2009.07.032.

López C, Corona A, Rincón JE. 1997. Relación entre parámetros biométricos y peso seco en insectos acuáticos depredadores de Venezuela. Rev Biol Trop. 44(3):641–643.

MacLean SF. 1973. Life Cycle and Growth Energetics of the Arctic Crane fly Pedicia hannai antenatta. Oikos. 24(3):436. doi:10.2307/3543820.

Mährlein M, Pätzig M, Brauns M, Dolman AM. 2016. Length–mass relationships for lake macroinvertebrates corrected for back-transformation and preservation effects. Hydrobiologia. 768(1):37–50. doi:10.1007/s10750-015-2526-4.

Maier KJ, Kosalwat P, Knight AW. 1990. Culture of Chironomus decorus (Diptera: Chironomidae) and the Effect of Temperature on its Life History. Environ Entomol. 19(6):1681–1688. doi:10.1093/ee/19.6.1681.

Merritt RW, Ross DH, Larson GJ. 1982. Influence of Stream Temperature and Seston on the Growth and Production of Overwintering Larval Black Flies (Diptera: Simuliidae). Ecology. 63(5):1322–1331. doi:10.2307/1938860.

Méthot G, Hudon C, Gagnon P, Pinel-Alloul B, Armellin A, Poirier A-MT. 2012. Macroinvertebrate size–mass relationships: how specific should they be? Freshw Sci. 31(3):750–764. doi:10.1899/11-120.1.

Miserendino ML. 2001. Length-mass relationships for macroinvertebrates in freshwater environments of Patagonia (Argentina). Ecol Austral. 11:3–8.

Morin A, Back C, Chalifour A, Boisvert J, Peters RH. 1988. Effect of Black Fly Ingestion and Assimilation on Seston Transport in a Quebec Lake Outlet. Can J Fish Aquat Sci. 45(4):705–714. doi:10.1139/f88-085.

Morin A, Constantin M, Peters RH. 1988. Allometric Models of Simuliid Growth Rates and Their Use for Estimation of Production. Can J Fish Aquat Sci. 45(2):315–324. doi:10.1139/f88-037.

Neveu A. 1977. Ecologie des larves d’Athericidae (Diptera, Brachycera) dans un ruisseau des Pyrenees Atlantiques. II. Production. Comparaison de differentes methodes de calcul. Ann d’Hydrobiologie. 8:45–66.

Nolte U. 1990. Chironomid biomass determination from larval shape. Freshw Biol. 24(3):443–451. doi:10.1111/j.1365-2427.1990.tb00723.x.

Oliphant ZH, Hyslop EJ. 2020. Biomass, Productivity, and Biomass Turnover (P/B) Ratios of Benthic Macroinvertebrates in High Elevation Ponds in St. Ann, Jamaica (West Indies). Caribb J Sci. 50(2). doi:10.18475/cjos.v50i2.a11.

Potter DWB, Learner MA. 1974. A study of the benthic macro-invertebrates of a shallow eutrophic reservoir in South Wales with emphasis on the Chironomidae (Diptera); their life histories and production. Arch für Hydrobiol. 74:186–226.

Ribeiro GC. 2008. Phylogeny of the Limnophilinae (Limoniidae) and early evolution of the Tipulomorpha (Diptera). Invertebr Syst. 22(6):627. doi:10.1071/IS08017.

Schröder P. 1987. Biomasseparameter der Larvenstadien mitteleuropäischer Kriebelmückenarten (Diptera: Simuliidae). Arch für Hydrobiol. 77(1):97–115.

Searle SR, Speed FM, Milliken GA. 1980. Population Marginal Means in the Linear Model: An Alternative to Least Squares Means. Am Stat. 34(4):216–221. doi:10.1080/00031305.1980.10483031.

Sephton TW, Paterson CG. 1986. Production of the chironomid Procladius bellus in an annual drawdown reservoir. Freshw Biol. 16(6):721–733. doi:10.1111/j.1365-2427.1986.tb01013.x.

Shadrin N V., Belyakov VP, Bazhora AI, Anufriieva E V. 2019. Does salinity affect body proportions and “size/mass” ratios of highly halotolerant Baeotendipes noctivagus larvae (Diptera, Chironomidae)? Oceanol Hydrobiol Stud. 48(4):305–315. doi:10.2478/ohs-2019-0028.

Shahbaz-Gahroee S, Aazami J, Aghamohammadi A, Rico A, Sumon KA. 2021. Length-mass relationships for macroinvertebrates in the Choghakhor international wetland, Iran. Biologia (Bratisl). 76(2):645–653. doi:10.2478/s11756-020-00585-w.

Smock LA. 1980. Relationships between body size and biomass of aquatic insects. Freshw Biol. 10(4):375–383. doi:10.1111/j.1365-2427.1980.tb01211.x.

Stockner JG. 1971. Ecological Energetics and Natural History of Hedriodiscus truquii (Diptera) in Two Thermal Spring Communities. J Fish Res Board Canada. 28(1):73–94. doi:10.1139/f71-012.

Towers DJ, Henderson IM, Veltman CJ. 1994. Predicting dry weight of New Zealand aquatic macroinvertebrates from linear dimensions. New Zeal J Mar Freshw Res. 28(2):159–166. doi:10.1080/00288330.1994.9516604.

de Vienne DM. 2016. Lifemap: Exploring the Entire Tree of Life. PLOS Biol. 14(12):e2001624. doi:10.1371/journal.pbio.2001624.

Wiegmann BM, Trautwein MD, Winkler IS, Barr NB, Kim J-W, Lambkin C, Bertone MA, Cassel BK, Bayless KM, Heimberg AM, et al. 2011. Episodic radiations in the fly tree of life. Proc Natl Acad Sci. 108(14):5690–5695. doi:10.1073/pnas.1012675108.

Wotton RS. 1978. Life-histories and production of blackflies (Diptera: Simuliidae) in moorland streams in upper Teesdale, Northern England. Arch Hydrobiol. 83:232–250.
