# Supplementary figures and images for: Length–mass allometries of the larvae of aquatic dipterans: differences between taxa, morphological traits, and methods
Source: J Insect Sci. 2024 Feb 17;24(1):10. doi: 10.1093/jisesa/ieae012 (PMC10874217; doi:10.1093/jisesa/ieae012)

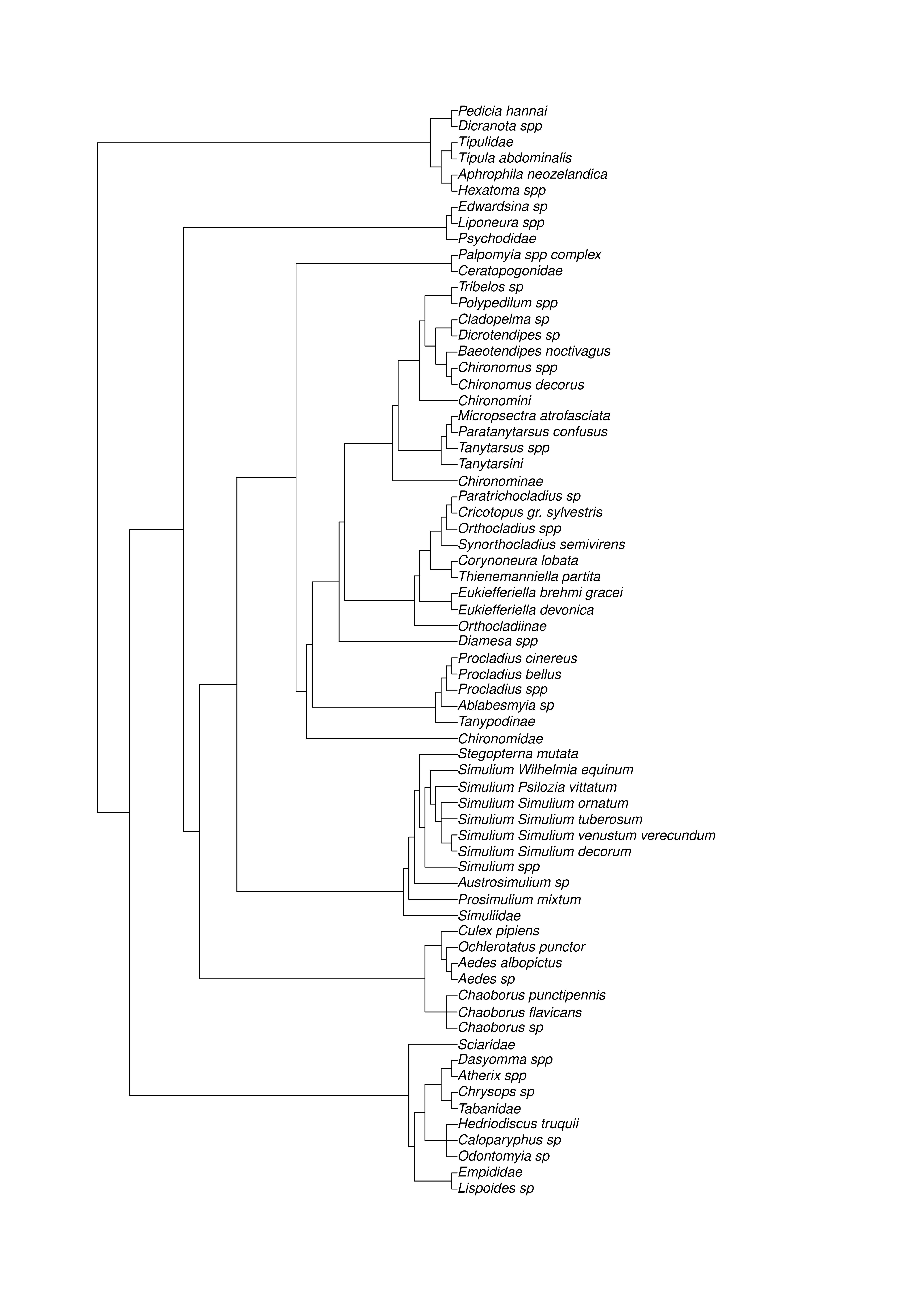

Supplement: ieae012_suppl_Supplementary_Material [file ieae012_suppl_supplementary_material.zip › FigS1_DipteraPhylogeny.tif]
